# Therapeutic Modulation of Meningeal Lymphatics: A Systematic Review of Preclinical Evidence Across Neurological Disorders

**DOI:** 10.1007/s10571-025-01611-8

**Published:** 2025-10-16

**Authors:** Sedef Kollarik, Sophie Katharina Humer, Carmen Elena Zurfluh, Epameinondas Gousopoulos, Nicole Lindenblatt

**Affiliations:** 1 Department of Plastic Surgery and Hand Surgery, University Hospital Zurich, Zurich, Switzerland; 2 Department of Visceral Surgery and Transplantation, University Hospital Zurich, Zurich, Switzerland; 3 Faculty of Medicine, University of Zurich, Zurich, Switzerland

**Keywords:** Meningeal lymphatic vessels, Lymphatic drainage, Deep cervical lymph node, Neurodegeneration, Neuroinflammation, Brain disease

## Abstract

Meningeal lymphatic vessels (MLVs) have emerged as critical modulators of cerebral homeostasis, immune surveillance, and metabolic clearance. Their dysfunction is increasingly implicated in the pathogenesis of neurodegenerative and neuroinflammatory conditions. This systematic review aimed to synthesize current preclinical evidence on the therapeutic modulation of MLVs across animal models of neurological disease, focusing on pathological, behavioral, and immunological outcomes. We conducted the literature search in the PubMed, Embase, Web of Science, and Scopus databases in accordance with PRISMA guidelines and included peer-reviewed, controlled preclinical studies investigating interventions aimed at enhancing meningeal lymphatic drainage in neurological disease models. Risk of bias was assessed using Covidence’s quality assessment template, supported by the SYRCLE Risk of Bias tool. Given the heterogeneity of models and interventions, a qualitative synthesis was performed. Therapeutic strategies were consistently associated with improved MLV structure and function, enhanced clearance of neurotoxic proteins, reduced neuroinflammation, and improved cognitive and motor performance across the disease models. Thus, enhancing meningeal lymphatic drainage represents a promising preclinical therapeutic approach for a wide spectrum of neurological conditions. Future research should aim to standardize methodologies, explore sex- and age-specific effects, and accelerate translation into human trials.

## Introduction

The lymphatic system; a network of vessels, nodes, ducts, and various lymphoid organs, extends throughout the body and plays a crucial role in maintaining tissue homeostasis and immune function by absorbing excess fluid and transporting the immune cells, respectively (Secker and Harvey 2015). However, it was a common belief until recently that the brain lacked a conventional lymphatic system. Initial hypotheses suggested that the central nervous system (CNS) primarily relied on cerebrospinal fluid (CSF) circulation (Brinker et al. 2014; Redzic et al. 2005) and the blood–brain barrier (Deane et al. 2003) in metabolite balance and clearance.

In 1787, an Italian anatomist, Paolo Mascagni, first mentioned the existence of lymphatics in the brain in his published work, *Vasorum lymphaticorum corporis humani historia et ichnographia (*Bucchieri et al. 2015*)*. However, for many years thereafter, his observations were largely forgotten. Over the last century, the advancements in imaging and molecular biology have revolutionized our understanding of the brain’s waste-clearance systems. Recent discoveries have highlighted a unique extracellular space clearance system in the brain, known as the glymphatic system facilitated by aquaporin-4 (AQP4) water channels on the vascular side of astrocytic endfeet (Iliff and Nedergaard 2013; Iliff et al. 2012; Jessen et al. 2015; Nedergaard 2013). In parallel, researchers have identified functional meningeal lymphatic vessels (MLVs) in the dural sinuses that extend throughout the entire meningeal compartment, regulating immune cell recirculation and facilitating drainage of soluble waste products to deep cervical lymph nodes (dCLNs; Aspelund et al. 2015; Louveau et al. 2015; Ma et al. 2019, 2017). The MLVs have also been observed in humans and nonhuman primates through contrast-enhanced magnetic resonance imaging (MRI) and immunohistochemical analysis of postmortem tissue samples (Absinta et al. 2017).

Different than the lymphatic vessels of peripheral tissues, MLVs develop during the postnatal period (Antila et al. 2017; Balint et al. 2019), and they are similarly dependent on vascular endothelial growth factor-C (VEGF-C) and vascular endothelial growth factor receptor-3 (VEGFR-3) for lymphangiogenesis (Antila et al. 2017). MLVs further express several key lymphatic markers, including lymphatic vessel endothelial hyaluronan receptor 1 (LYVE1), Prospero homeobox protein 1 (PROX1), podoplanin, and C-C motif chemokine ligand 21 (CCL21). Of these, the VEGFC-/VEGFR3 signaling pathway, plays a pivotal role in the development and plasticity of MLVs, thereby serving as a potential target for various therapeutic strategies (Absinta et al. 2017; Aspelund et al. 2015; Kim et al. 2025; Louveau et al. 2015).

Age-related structural and functional changes in MLVs compromise their ability to efficiently clear waste products and contribute to the accumulation of macromolecules (Da Mesquita et al. 2021a, b; Da Mesquita et al. 2018). In neurodegenerative disorders, particularly those marked by progressive toxic accumulation of pathological protein aggregates, like in Alzheimer’s and Parkinson’s, dysfunction of MLVs exacerbates the pathology by further accumulating the neurotoxic substances (Da Mesquita et al. 2021a, b; Louveau et al. 2016; Patel et al. 2019). Consequently, improving or restoring lymphatic drainage offers a promising therapeutic strategy, helping to prevent or delay neurodegenerative and neuroinflammatory conditions (Laaker and Fabry 2021; Tavares and Louveau 2021).

Currently, there is no clinically established or scientifically proven method for reliably enhancing meningeal lymphatic drainage in humans. However, several synergistic strategies are being explored, including pharmacological, non-pharmacological, genetic, and immunological approaches, with research primarily being confined to animal models (Formolo et al. 2023; Gao et al. 2025; Melloni et al. 2023).

Building on this emerging clinical interest, a novel surgical approach was aimed at increasing drainage of cerebrospinal and interstitial fluid from the brain through the deep cervical lymph nodes by performing deep cervical lymphovenous anastomosis (LVA) in patients with cognitive impairment (Xie et al. 2024). This technique, adapted from established super-microsurgical treatments for lymphedema, is grounded in two key principles: the “catchment effect,” where enhancing lymphatic drainage in a targeted anatomical region improves local clearance (Lin et al. 2009); and the “systemic effect,” in which modulating one part of the lymphatic system leads to broader improvements throughout the interconnected lymphatic network (Kukreja-Pandey et al. 2023). As a result, Dr. Xie observed significant improvements in language, cognition, motor function, and behavior following the lymphatic reconstruction in the patients. In another case study, they performed LVA surgery in an Alzheimer’s disease patient, meeting biological diagnostic criteria, and showed postoperative improvements in cognitive function, mood, and brain imaging markers, suggesting the procedure’s potential as a novel treatment approach (X. Li et al. 2024a, b).

Furthermore, a number of clinical trials are currently in progress and actively recruiting, translating preclinical findings on meningeal lymphatics and neurological disorders into early-stage clinical phases. These include, among others, studies investigating the effects of monoclonal antibodies (Ofatumumab) on meningeal lymphatic drainage in patients with demyelinating diseases (NCT05414487), as well as exploratory trials assessing Modified Deep Cervical Lymphovenous Anastomosis (LVA) in Alzheimer’s and Parkinson’s disease (NCT06852352), and Deep Cervical Lymphovenous Bypass (LVB) in Alzheimer’s disease (NCT0644897).

Given the limited availability of clinical studies in this emerging field, this systematic review aims to comprehensively examine the role and therapeutic potential of MLVs in alleviating neurodegenerative and neuroinflammatory conditions in animal models based on the current state of research, while also providing a solid foundation for potential future clinical investigations.

## Methods

### Search Strategy/Literature Search

This systematic review was registered with PROSPERO (ID CRD42024582229) under the title: “Role of Meningeal Lymphatic Drainage in Animal Models of Brain Disease and Disorders: A Systematic Review.”

On September 5, 2024, we conducted a systematic literature search according to the Preferred Reporting Items for Systematic Reviews and Meta-Analyses (PRISMA) guidelines (Page et al. 2021). The search was performed online on the PubMed, Embase, Web of Science and Scopus databases using the following search terms and Boolean operators: (animal) AND (drainage) AND (meningeal lymphatic) AND ((neurodegeneration) OR (neuroinflammation) OR (brain disease)).

The initial search yielded a total of 554 results (PubMed: 62, Web of Science: 204, Embase: 203 and Scopus: 85). To ensure the quality and efficiency of our systematic review, we utilized Covidence (Covidence systematic review software, Veritas Health Innovation, Melbourne, Australia) to support both the screening process and data extraction. After initially collecting the results in EndNote, we exported the sources to Covidence, where duplicates were removed, leaving 306 papers for title and abstract screening. In addition to this automated process, we identified and removed 12 duplicates manually.

### Study Selection

**Fig. 1:**
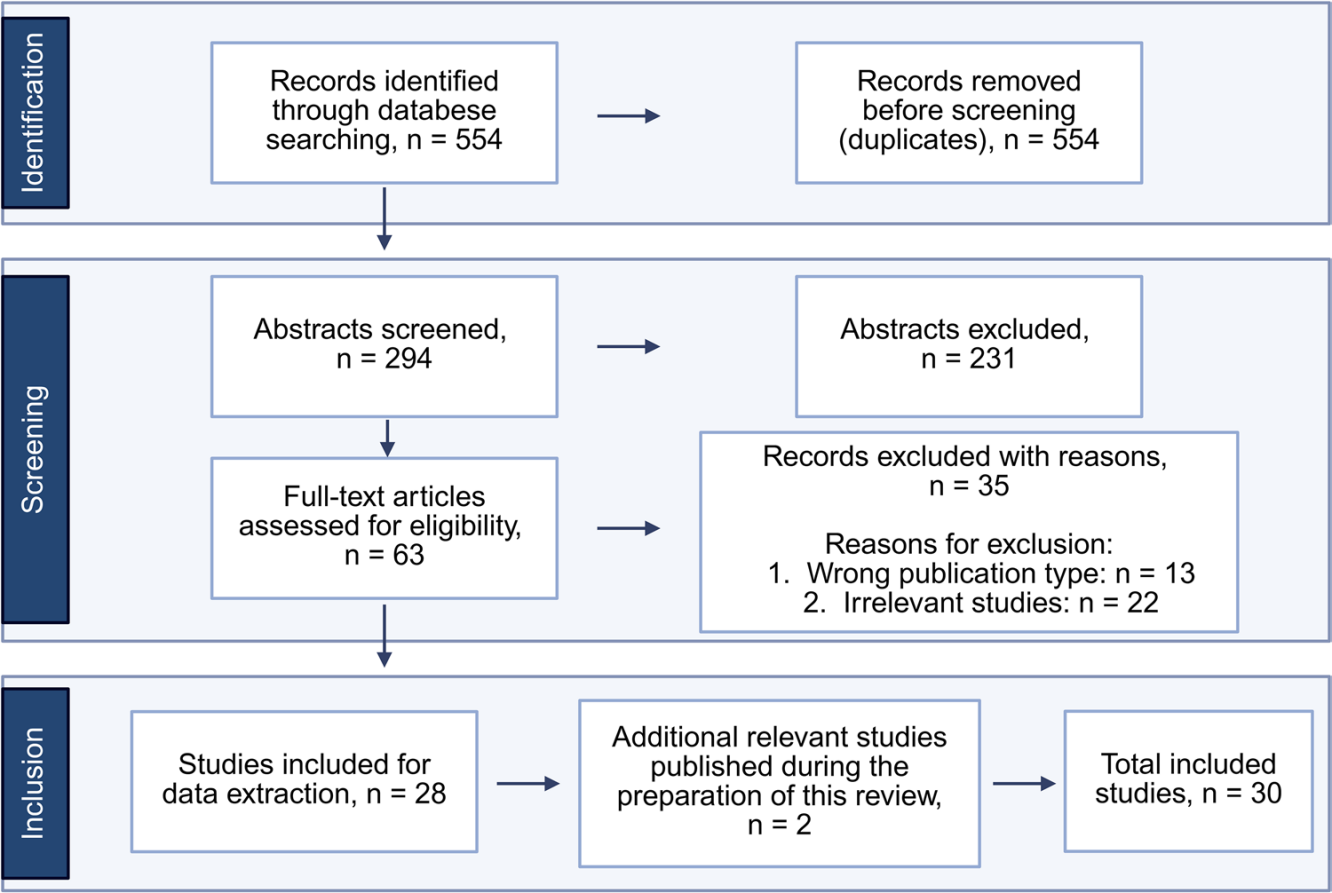
Flowchart of the study search and selection process

The studies included in the screening process were evaluated in two steps. In the first step, the two main reviewers (S.K. and S.H.) independently assessed eligibility of the titles and abstracts of the remaining 294 studies, of which 63 were confirmed for further assessment. In cases of conflict (8 in total) during the title and abstract screening, a third reviewer (C.E.Z.) was consulted to resolve these discrepancies. In the second step, the selected studies were assessed based on their full text. After resolving three additional conflicts, a final total of 28 studies were included in this systematic review. Furthermore, two relevant studies published during the preparation of this review were identified and included, bringing the final number of included studies to 30 (Fig. 1).

We included original, peer-reviewed, preclinical studies that investigated interventions aimed at enhancing meningeal or cerebral lymphatic drainage in mammalian models with comparable lymphatic anatomy to humans. Eligible populations comprised rodents and other relevant mammals of any age or sex, with induced neurological disorders. Interventions encompassed any surgical, pharmacological, genetic, biomaterial-based, or non-invasive approaches directly targeting MLV structure or function.

We excluded ex vivo, in vitro, in silico, and clinical studies; animal models without a meningeal lymphatic system comparable to humans; non-neurological disease models; observational studies without interventions targeting meningeal lymphatic drainage; and non-experimental publications such as reviews, editorials, and expert opinions. Additionally, only peer-reviewed, English journal articles with a separate control group were considered. Studies with control groups that did not match the experimental group, lacked methodological rigor, or had unclear outcome measures were excluded.

### Data Extraction

**Fig. 2:**
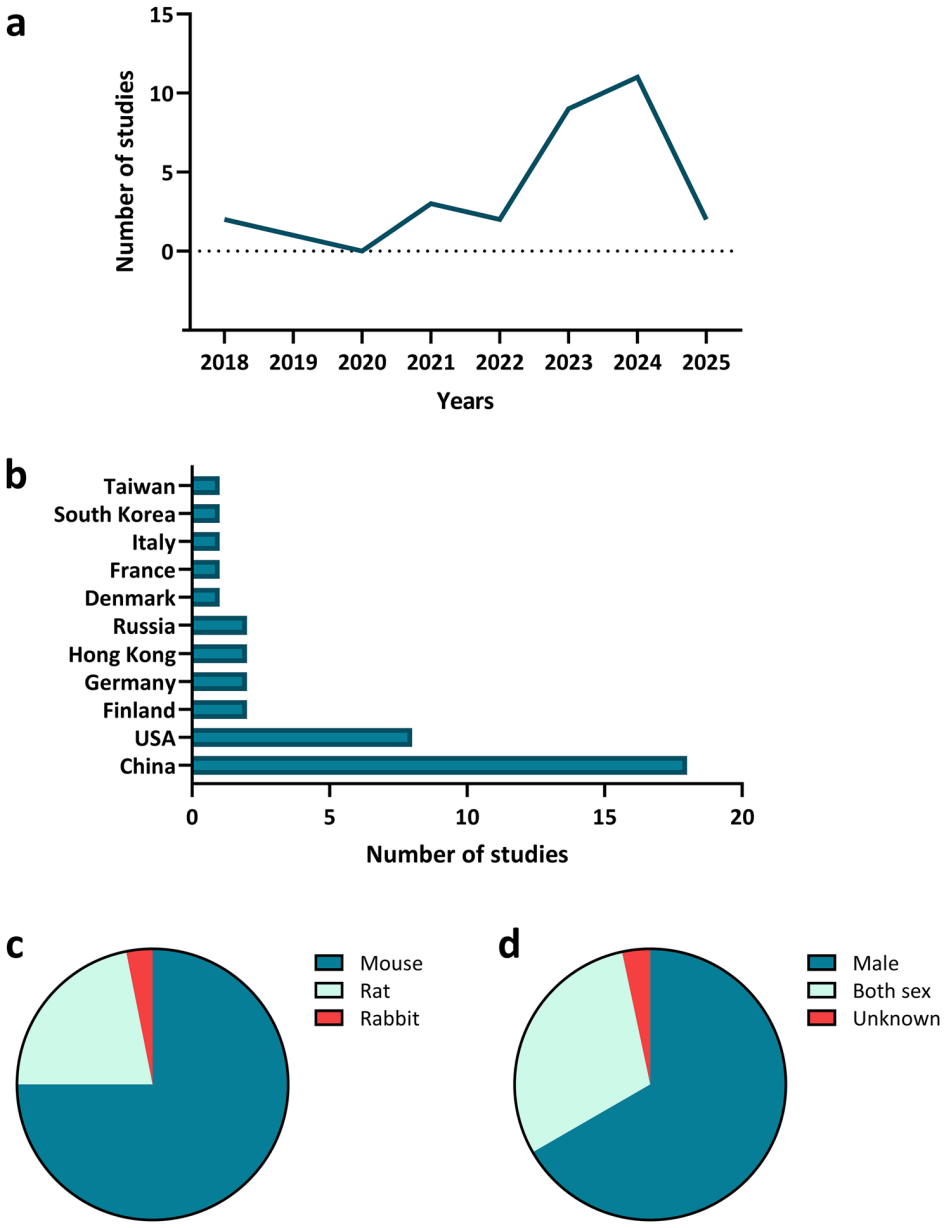
Overview of included studies: distribution by year of publication, country, animal species, and sex. This figure summarizes the characteristics of the 30 preclinical studies included in this systematic review. **a** Number of studies published per year, showing trends over time. **b** Number of studies conducted by country, highlighting the geographic distribution of research activity. **c** Percentage distribution of animal species used in the studies (mouse, rat, rabbit). **d** Proportion of male and female animals used, indicating the sex balance in experimental designs

Two independent reviewers (S.K. and S.H.) performed the data extraction of the included studies using Covidence. For each study, the following data were extracted: (1) Study identification: title, author, email, journal, year of publication, institution, funding source, address, country; (2) Methods of the respective animal intervention study; (3) Population: differences between groups, number of groups, species, disease model, sex, age, reasons for withdrawal; (4) Interventions; (5) Outcomes; (6) Results with the respective statistical data. Once the data extraction in Covidence was completed, the two main reviewers compared their findings and resolved any discrepancies through a consensus process. The agreed-upon data was then exported to Excel for further processing. Study demographics are summarized in Fig. 2.

### Quality Assessment

The quality of the 30 studies was assessed using Covidence’s quality assessment template, with the support of the SYRCLE Risk of Bias tool (Higgins et al. 2024). Each of the 10 risks of bias domains (selection, performance, detection, attrition, reporting bias, and other sources of bias) was assessed as unclear, low, or high for every study (Table 1). Any potential disagreements were resolved through discussion between the two primary reviewers during the comparison process.

**Table 1:**
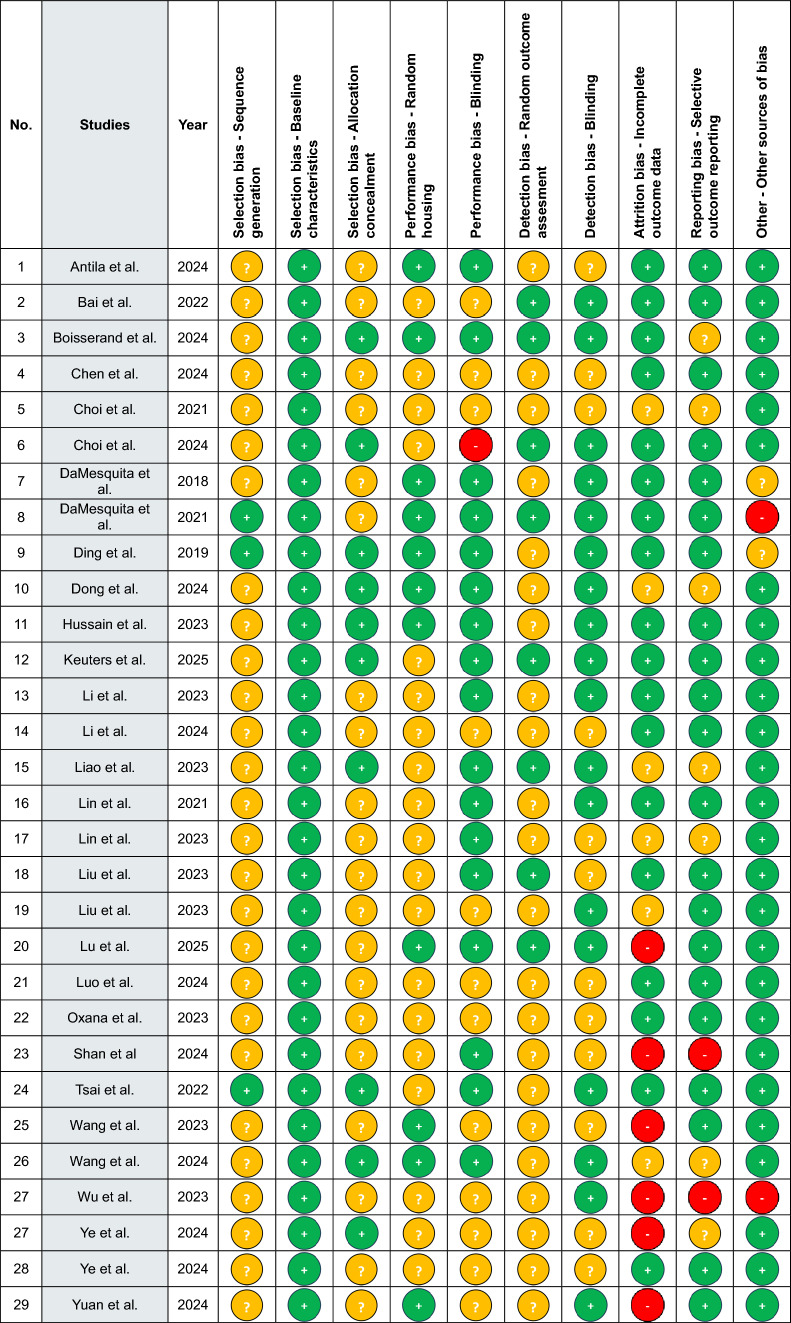
Quality assessment of the screened studies included in this review

This table presents the quality assessment of the 30 preclinical studies included in this review, evaluated using SYRCLE’s risk-of-bias tool. Each study was assessed across key domains including selection bias, performance bias, detection bias, attrition bias, reporting bias, and other potential sources of bias. Risk of bias was classified for each category as *low risk (green)*, *high risk (red)*, or *unclear risk (yellow)* based on the reported methodology

### Statistical Analysis

Due to the substantial heterogeneity of the neurological and neurodegenerative conditions studied, as well as the variety of experimental models used within each condition, statistical meta-analysis was not appropriate. Therefore, a qualitative synthesis of the findings was conducted. Figures in this study were created using GraphPad Prism, Microsoft Excel, Microsoft Word and BioRender.

## Results

### Effect of Meningeal Lymphatic Drainage in Several Neurodegenerative and Neurological Conditions

**Fig. 3:**
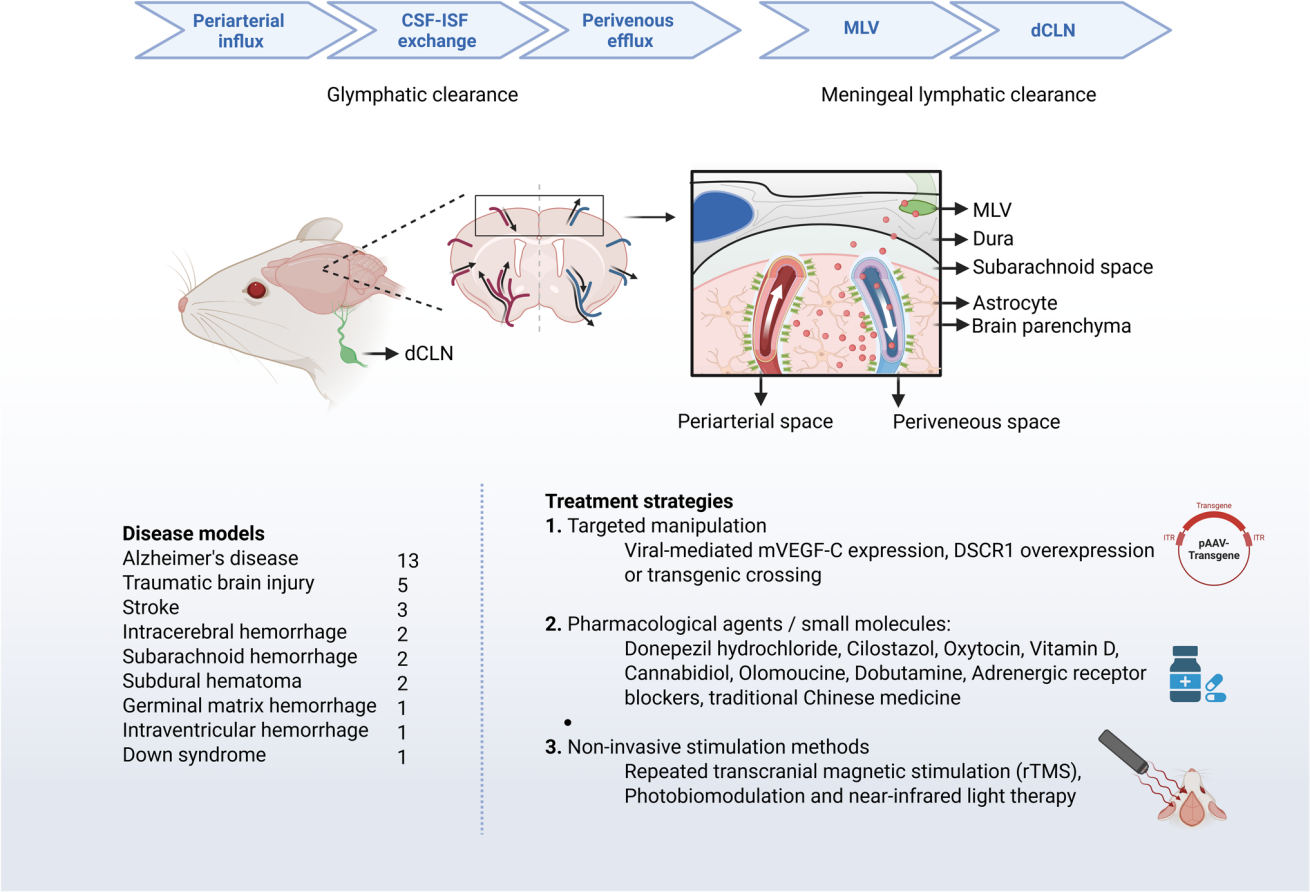
Graphical overview of the preclinical studies included in this review, illustrating the treatments and interventions tested across different animal models. Studies included Alzheimer’s disease, traumatic brain injury, stroke, intracerebral, subarachnoid, germinal matrix and intraventricular hemorrhages, subdural hematoma, and down syndrome. Various treatment methods were used to enhance meningeal lymphatic drainage, including VEGF-C administration, light-based therapies, pharmacological agents, genetic interventions, biomaterial-based delivery systems, and noninvasive stimulation methods. The outcome measures were mainly focused on histopathological changes, behavioral and cognitive performance, imaging-based assessment of lymphatic drainage, and analysis of neuroinflammatory markers. The figure was generated with BioRender (BioRender.com)

Our research strategy revealed 30 animal studies (mouse, *n* = 24; rat, *n* = 7; rabbit, *n* = 1) on the effect of meningeal lymphatic drainage under various treatment options in some of the key neurodegenerative and neurological conditions. These conditions were Alzheimer's disease (AD), traumatic brain injury (TBI), stroke, intracerebral, subarachnoid, germinal matrix and intraventricular hemorrhages, subdural hematoma as well as Down syndrome (Fig. 3).

The results are organized into three sections based on disease context: AD (13 studies), TBI (5 studies), and other neurological comorbidities combined due to the smaller number of studies in each category (1–3 studies per condition), as illustrated in Fig. 2.

#### Alzheimer’s Disease

AD, which is the most common form of dementia and of the neurological disorders most closely associated with meningeal lymphatic dysfunction (Rego et al. 2023), unsurprisingly stands as the most researched disorder in this field. Studies included in this review utilized a diverse array of AD models, such as 5XFAD, APP/PS1, APPSWE, and bilateral injection of amyloid-beta (Aβ) into the hippocampus, to evaluate the impact of meningeal lymphatic drainage enhancement therapies on disease outcomes.

##### Effects of MLV Drainage Enhancement on Disease Pathology

Deposition of Aβ in the brain is one of the histopathological hallmarks of AD (Braak and Braak 1991; Chen et al. 2017; Haass and Selkoe 1993; Selkoe 2001), and accumulation of these misfolded proteins is believed to result from an imbalance between their production and clearance (Mawuenyega et al. 2010; Sadigh-Eteghad et al. 2015). Thus, enhancing the drainage capability of MLVs may improve the clearance of these toxic proteins. Eleven studies on animal models of AD included in this systematic review revealed that interventions targeting meningeal lymphatic function promoted lymphatic drainage and reduced the amyloid burden in the brain. Overexpression of DSCR1 in 5XFAD mice significantly reduced hippocampal Aβ plaque load, indicating a protective effect against Aβ pathology (Choi et al. 2021). While, injection of recombinant human VEGF-C alone into the cisterna magna (i.c.m.) or lateral ventricle (i.c.v.) did not significantly affect Aβ levels or plaque load (Antila et al. 2024; Da Mesquita et al. 2018), its combination with passive mAb158 (the murine chimeric analogs of Aducanumab) immunotherapy enhanced Aβ clearance (Da Mesquita et al. 2021a, b). Moreover, other interventions, such as oral administration of Yuanzhi powder and borneol, intranasal administration of oxytocin, repeated transcranial magnetic stimulation (rTMS), photo-biomodulation and near-infrared light therapy on the skull surface, and dCLN delivery of donepezil hydrochloride + cilostazol via lyotropic liquid crystalline (LLC) implants effectively reduced Aβ deposition by improving its clearance through enhanced meningeal lymphatic drainage (J. Li et al. 2024a, b; Lin et al. 2021; Oxana et al. 2023; Shan et al. 2024; Wang et al. 2024; Wu et al. 2023; C. Ye et al. 2024a, b; T. Ye et al. 2024a, b). Among the included studies, one utilized a surgical approach, cranial bone maneuver (CBM), to enhance meningeal lymphatic drainage, and observed a significant reduction in amyloid plaque density in the hippocampus and thalamus of 5xFAD mice, but not in the cortex (Lu et al. 2025).

##### Behavioral Outcomes Following MLV Modulation Therapies

**Fig. 4:**
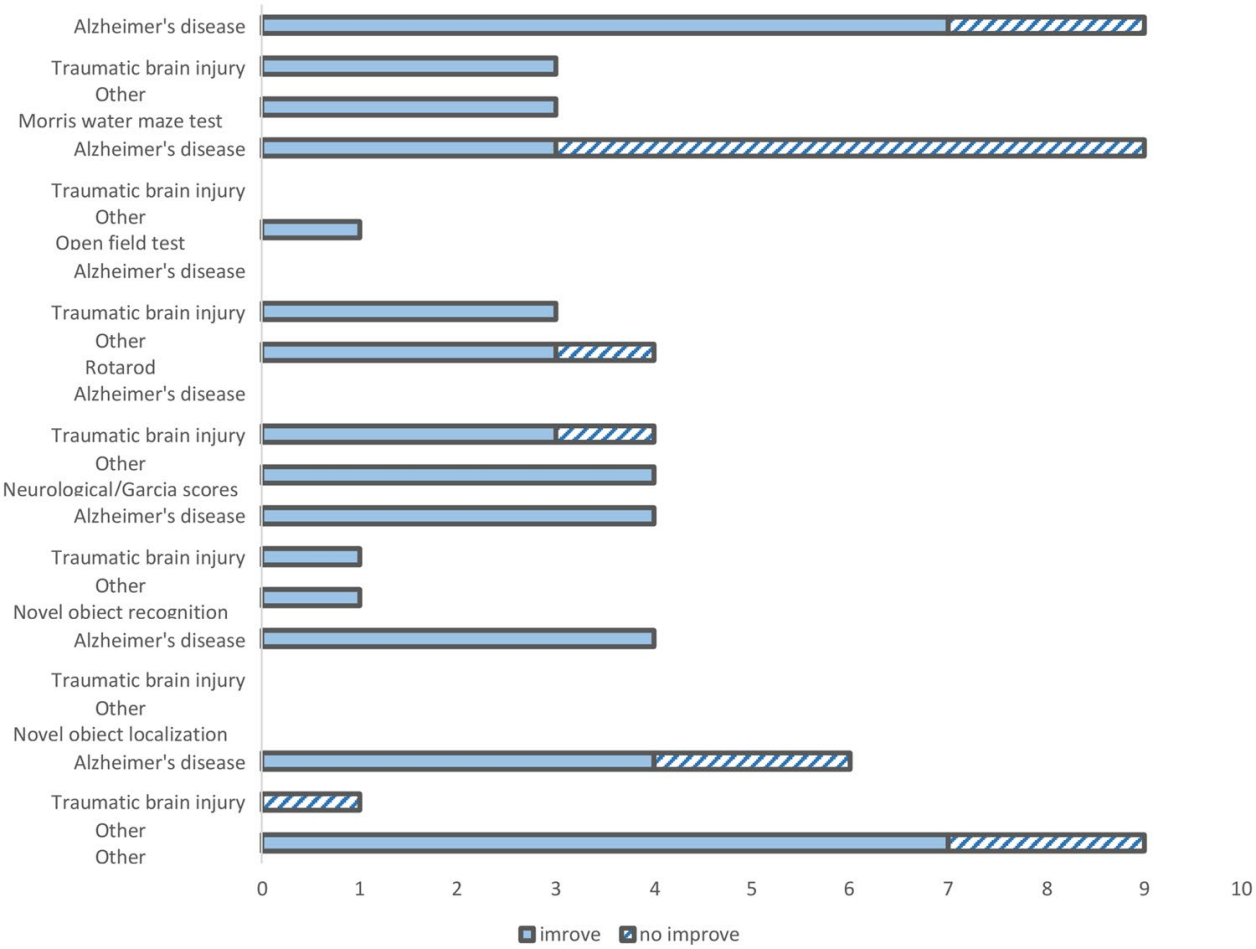
Summary of behavioral improvements reported across disease models and behavioral tests. This figure shows the number of studies that reported behavioral improvements following interventions to enhance meningeal lymphatic drainage, categorized by disease area: Alzheimer’s disease (AD; *n* = 13 studies), traumatic brain injury (TBI; *n* = 5 studies), and a combined “other” category (*n* = 1–3 studies each, including stroke, hemorrhages, subdural hematoma, and Down syndrome). Behavioral tests that were applied in only 1–2 studies were grouped together under “Other” and include the hanging wire test, mesh hanging test, negative geotaxis, righting reflex, foot fault test, laterality index, Y maze, beam walk score, round stick balance score, and string suspension grip score. Solid blue bars represent the number of studies that showed improvement after treatment, while the lined bars indicate the number of studies that reported no improvement

A range of behavioral tests [Morris water maze (MWM)], open field test, novel object/location recognition tests (NORT, NOLT, NLR), Y maze test, Barnes maze test, contextual fear conditioning (CFC), and nest building) were used to evaluate cognitive, spatial learning, memory, anxiety-like behavior, and fine motor functions (Fig. 4). MWM and open field tests were the most commonly used methods to assess behavioral changes following meningeal lymphatic interventions in AD.

Studies reported improvement in spatial learning and memory assessed by the MWM test in treatment groups receiving near-infrared light treatment, DSCR1 upregulation or crossing with DSCR1 mice, Yuanzhi powder (only with high-dose), donezepil, borneol and oxytocin, indicating enhanced cognitive performance in these AD models (Choi et al. 2021; J. Li et al. 2024a, b; Lin et al. 2021; Shan et al. 2024; Wang et al. 2024; Wu et al. 2023; C. Ye et al. 2024a, b). However, interventions such as prolonged mAducanumab or mAb158 antibody treatments and viral-mediated expression of mVEGF-C did not yield significant improvements in MWM performance (Da Mesquita et al. 2018; Da Mesquita et al. 2021a, b). Enhanced performance in the open field test, reflected by increased center entries and reduced anxiety-like behaviors, was observed only in animals treated with donezepil, oxytocin and near-infrared light (Shan et al. 2024; Wang et al. 2024; C. Ye et al. 2024a, b). Similarly, the Barnes Maze test, another behavioral assay for spatial learning and memory, demonstrated that CBM significantly decreased escape latency during the acquisition phase in 5xFAD mice (Lu et al. 2025). Studies showed that recognition memory, as measured by the NORT, and spatial memory, assessed by the NOLT, both improved following enhanced meningeal lymphatic drainage (J. Li et al. 2024a, b; Lin et al. 2021; Lu et al. 2025; Wang et al. 2024; C. Ye et al. 2024a, b). Additionally, one study observed improvements in nest-building behavior but not in contextual CFC performance (Shan et al. 2024), and another study demonstrated enhancements in spatial working memory assessed by Y maze test (C. Ye et al. 2024a, b).

##### Lymphangiogenic and Morphological Responses of MLVs to Therapeutic Modulation

The structure of MLVs alters by aging and diseases associated with the CNS (Liao et al. 2023; Ma et al. 2017). Studies included in this review investigated the changes in the diameter and length of MLVs, volume and weight of dCLNs and markers of lymphangiogenesis following therapeutic interventions. Treatment with Adeno-associated viruses (AAV) encoding VEGF-C consistently induced robust and sustained dural lymphatic vessel (dLV) expansion regardless of the injection route (i.c.v. or i.c.m. delivery) across two AD mouse models: APdE9 (analyzed at 9 months to assess early pathology and at 15 months to test effects on established amyloid plaques) and 5xFAD (analyzed at 4.5 months to confirm findings in an aggressive AD model) mice (Antila et al. 2024). The lymphatic defects that are apparent in 5XFAD mice by 5 months are reversed in DSCR1/5XFAD mice by preserving or enhancing the MLV morphology (Choi et al. 2021). mVEGF-C treatment failed to make differences in the morphology of MLVs (Da Mesquita et al. 2018), however its combination with anti-Aβ antibody mAb158 demonstrated lymphatic vessel expansion around the transverse sinus (TS; Da Mesquita et al. 2021a, b). The reduced diameter and coverage of MLVs in TS of 6-month-old APP/PS1 mice were significantly improved by 8 weeks of high-dose Yuanzhi powder treatment (J. Li et al. 2024a, b). In another study with the APP/PS1 model, intranasal administration of oxytocin at 11 months did not significantly alter meningeal lymphatic vessel diameter, indicating minimal impact on vessel constriction or dilation; however, it restored VEGF-C expression, leading to increased levels of LYVE-1 and Prox1 at both the protein and mRNA levels, thereby promoting lymphangiogenesis and structural remodeling of MLVs (C. Ye et al. 2024a, b). In a bilateral Aβ42 hippocampal injection model simulating Alzheimer's pathology, oral administration of borneol, either as a preventive or therapeutic strategy, effectively restored the expression of LYVE-1 and FOXC2 and reversed dysregulation of valve-related transcription factors, while oral and subcutaneous donepezil–cilostazol formulations reduced lymphatic vessel coverage in meninges and dCLNs (Shan et al. 2024; T. Ye et al. 2024a, b). In 5xFAD (6 months) and APP/PS1 (11 months) AD mouse models, transcranial 808 nm light treatment increased branch length and number, enlarged the diameter of MLVs, and enhanced the lymphangiogenesis as evidenced by increased LYVE-1 area in meninges(Wang et al. 2024). CBM-performed 5xFAD mice showed a significant increase in LYVE-1–positive area fraction of MLVs in the regions of the superior sagittal sinus and transverse sinus (Lu et al. 2025).

##### Restoring CSF and Meningeal Lymphatic Drainage in Pre-clinical AD Models

MLV drainage imaging methods involve the use of tracers (e.g., fluorescent or contrast agents) injected to visualize and quantify the MLV drainage properties by tracking tracer movement from the cerebrospinal fluid to the cervical lymph nodes using imaging techniques such as MRI, two-photon microscopy, or near-infrared fluorescence imaging. Across Alzheimer’s disease models, several interventions enhanced MLV clearance, though through diverse mechanisms. These included VEGF-C delivery, treatments with compounds such as Yuanzhi powder, oxytocin, Borneol, the surgical cranial CBM, and non-invasive strategies like rTMS, light stimulation, and photobiomodulation (Antila et al. 2024; J. Li et al. 2024a, b; Lin et al. 2021; Lu et al. 2025; Oxana et al. 2023; Wang et al. 2024; C. Ye et al. 2024a, b; T. Ye et al. 2024a, b) One of the studies observed the interventions to be more effective during sleep than wakefulness (Oxana et al. 2023). While most interventions demonstrated improved tracer clearance, reduced accumulation, or restored cerebral blood flow, outcomes were context-dependent, varying by disease model, timing of intervention, and outcome measure.

##### Therapeutic Targeting of MLVs and Its Modulation of Neuroinflammation in AD

Growing evidence highlights the critical role of immune processes in contributing to AD development and progression (Heneka et al. 2024). The analysis of key inflammatory markers provides insight into how impaired MLV drainage may exacerbate immune activation in the AD brain, and how therapeutic strategies aimed at restoring MLV function could help reverse these effects. Only five studies in this systematic review investigated the effects of MLV drainage therapies on neuroinflammation and immune responses in AD (Da Mesquita et al. 2021a, b; Lin et al. 2021; Lu et al. 2025; Wang et al. 2024; C. Ye et al. 2024a, b). Most of them reported reduced microglial activation, restored homeostatic gene expression, and downregulated pro-inflammatory pathways (Da Mesquita et al. 2021a, b; Lin et al. 2021; Wang et al. 2024; C. Ye et al. 2024a, b), however, CBM surgery demonstrated increased levels of microglial activation (Lu et al. 2025).

#### Traumatic Brain Injury

TBI is a leading cause of disability and death worldwide, with a rising global incidence and substantial socioeconomic burden (Robinson 2021); notably, brain trauma also causes severe deficits in meningeal lymphatic drainage that begin within hours of injury and persist for at least 1 month, potentially contributing to the chronic progression of TBI-related pathology (Bolte et al. 2020).

##### Impact of Enhanced MLV Function on Disease Pathology

TBI is frequently associated with the development of cerebral edema and disruption of the blood–brain barrier (BBB), both of which exacerbate secondary brain damage and influence neurological outcomes (Adelson et al. 1998; Shlosberg et al. 2010; Zima et al. 2024). In a ‘hit and run’ TBI mouse model, researchers observed an increase in the brain water content 30 min after the injury, and inhibition of adrenergic receptors [prazosin hydrochloride (10 µg/g), propranolol hydrochloride (10 µg/g) and atipamezole (1 µg/g), PPA; 3 times once a day] improved the brain fluid transport (Hussain et al. 2023). MRI scans performed at 1, 3, and 7 days post-TBI injury in rats demonstrated that ketoprofen and 9-*cis* retinoic acid significantly accelerated brain edema absorption by day 3 compared to DMSO controls, however VEGF-C treatment showed a trend toward improved edema absorption without reaching statistical significance (*p* = 0.087, Liao et al. 2023). Hydrogel-based VEGF-C treatment significantly reduced Evans blue (EB) leakage and preserved the tight junction protein ZO-1, indicating that these therapies attenuated BBB disruption (Lin et al. 2023).

##### Behavioral Changes Following MLV Modulation Therapies

Overall, treatments targeting the enhancement of MLV function were effective in improving behavioral outcomes in rodent models of TBI (Fig. 4). TBI mice treated with cannabidiol (CBD) for three times after trauma induction surgery exhibited a decreased neurological severity score (NSS), better motor coordination and balance in rotarod test, and improved spatial learning and memory in the MWM compared to vehicle-treated controls (Dong et al. 2024). After PPA treatment, behavioral results demonstrated significantly lower NSS, improved rotarod and string suspension performance, and enhanced spatial learning and memory in the Morris water maze with no differences in beam walk and round stick balance (Hussain et al. 2023). In a study, where ketoprofen, 9-*cis* retinoic acid (RA), and VEGF-C were administered to promote the proliferation of peripheral lymphatic vessels and thus, to enhance meningeal lymphatic function after TBI induction in rats, the neurological function score was considerably improved (Liao et al. 2023). Exogenous interleukin 33 (IL-33) significantly improved motor function as evidenced by mNSS and rotarod tests and alleviated TBI-induced deficits in short-term and spatial memory assessed by NORT and MWM tests (M. Liu et al. 2023a, b).

##### Lymphangiogenic and Morphological Responses of MLVs to Therapeutic Modulation

TBI induces transient lymphangiogenesis in the dorsal meningeal lymphatic vasculature, which is marked by increased LYVE-1 coverage, vessel diameter, and complexity (higher number of sprouts and loops) peaking at 1–2 weeks post-injury before largely returning to baseline by one month (Bolte et al. 2020). This suggests that a temporary structural remodeling response following TBI might be a compensatory mechanism for impaired drainage. The studies of this review also investigated morphological changes in the MLVs after TBI and evaluated the impact of treatments aimed at enhancing lymphatic drainage. In a moderate-to-severe closed-skull TBI mouse model, MLV diameter was slightly reduced, but increased significantly by adrenergic receptor inhibition treatment (Hussain et al. 2023). In a rat model of hydraulic shock-induced TBI, DMSO or H₂O showed decreased LYVE-1 area coverage and the effect was reversed by ketoprofen, 9-*cis*-RA, and VEGF-C treatment (Liao et al. 2023). In a moderate controlled cortical impact open-skull TBI mouse model, researchers observed enhanced LYVE-1 area coverage, increased loops and branches, and greater diameter, which diminished over time; however, hydrogel-based VEGF-C treatment sustained lymphangiogenesis long after TBI induction (Lin et al. 2023). A single IL-33 administration significantly restored and enhanced meningeal lymphatic vessel density evidenced by LYVE-1 area coverage in both the dorsal regions (confluence of the sinus and TS, with a more pronounced effect on the ipsilateral side) and at the basal occipital sinus (M. Liu et al. 2023a, b).

##### Restoring CSF and Meningeal Lymphatic Drainage Following TBI

Various therapeutic strategies to restore CSF drainage and meningeal lymphatic vessel (MLV) function following TBI proved to be promising as a therapy to mitigate secondary brain damage and support recovery. CBD treatment enhanced glymphatic solute drainage, improved AQP-4 polarization and CBF, and increased tracer clearance via MLVs and dCLNs compared to untreated TBI controls (Dong et al. 2024). Administration of PPA shortly after injury partially restored CSF influx (Hussain et al. 2023). Co-treatment with ketoprofen, 9-*cis* RA, and VEGF-C into the cisterna magna improved CSF drainage to brain tissue and dCLNs, reversing the drainage deficits observed post-injury (Liao et al. 2023). Hydrogel-based VEGF-C treatment significantly enhanced bead accumulation in dCLNs (Lin et al. 2023). Additionally, treatment with IL-33 effectively restored MLV drainage function after injury (M. Liu et al. 2023a, b).

##### Therapeutic Modulation of Neuroinflammation Following TBI

Neuroinflammation plays a central role in the secondary injury cascade following TBI, and recent studies have begun to explore how enhancing meningeal lymphatic drainage may modulate immune responses and attenuate inflammation in this context. All in all, these studies demonstrated that various therapeutic strategies (CBD treatment, adrenergic receptor blockade, anti-inflammatory and pro-lymphangiogenic agents, localized delivery of VEGF-C, and IL-33 administration) effectively reduce glial activation, pro-inflammatory signaling, and tau and Aβ pathology following TBI (Dong et al. 2024; Hussain et al. 2023; Liao et al. 2023; Lin et al. 2023; M. Liu et al. 2023a, b).

#### Other Neurological Conditions

Ischemic stroke, intracerebral hemorrhage (ICH), subarachnoid hemorrhage (SAH), germinal matrix hemorrhage (GMH), intraventricular hemorrhage (IVH), subdural hematoma (SDH), and neurodevelopmental disorders such as Down syndrome represent diverse neurological conditions characterized by varying degrees of vascular disruption, inflammation, and impaired clearance of interstitial and blood-derived waste from the brain (Motwani et al. 2022; Salvador et al. 2024).

##### Restoring MLV Function Alleviates Neuropathology in Ischemic and Hemorrhagic Brain Injury

Ischemic and hemorrhagic brain injuries are associated with a cascade of pathological changes that contribute to long-term neurological dysfunction. In two studies with models of ischemic stroke, volume of infarct lesions was significantly reduced after interventions (Bai et al. 2022; Boisserand et al. 2024). In another study, MRI-based analysis revealed that i.c.v. delivery of VEGF-C 14 or 35 days prior to ischemic stroke induction did not significantly affect brain infarct or edema volumes (Keuters et al. 2025). In ICH models, enhancing meningeal lymphatic drainage with VEGF-C significantly promoted hematoma clearance (Tsai et al. 2022). However, rTMS, while being beneficial for glymphatic clearance, did not influence BBB integrity (Y. Liu et al. 2023a, b). In a rat model SAH, VEGF-C treatment enhanced lymphatic drainage and significantly reduced glial activation, neuronal damage, and brain edema (Luo et al. 2024). In another study, 1 week after SAH, dobutamine treatment partially preserved neuronal morphology and increased neuronal survival, while significant neuronal damage was observed in control groups (Wang et al. 2022). In SDH models, both vitamin D and atorvastatin treatments accelerated hematoma absorption (Chen et al. 2024; Yuan et al. 2024). In a GMH model, vehicle-treated rats exhibited significant ventricular dilation, white matter loss, and cortical thinning, whereas olomoucine treatment mitigated these structural changes (Ding et al. 2019). In a postnatal day 4 (PD4) IVH model, photostimulation treatment significantly accelerated hematoma clearance, reducing hemorrhage size by 72.5% over 11 days and fully reversed vasogenic edema, as indicated by normalization of perivascular space size to sham levels (Li et al. 2023).

##### Behavioral Outcomes Following MLV Modulation Therapies

Three studies using the middle cerebral artery occlusion (MCAO) model of ischemic stroke [rat model with cranial bone transportation (CBT); using a 5 mm diameter bone flap, Bai et al. 2022 and the other two in mice receiving intra-cerebrospinal administration of an AAV expressing mouse full-length VEGF-C (Boisserand et al. 2024; Keuters et al. 2025)] demonstrated improved behavioral outcomes. The rat model showed enhanced motor recovery in the rotarod and Morris water maze tests (Bai et al. 2022), while VEGF-C-pretreated mice exhibited a longer hanging wire duration (Boisserand et al. 2024), improved NSS post-stroke (Boisserand et al. 2024; Keuters et al. 2025) and locomotor disturbances measured by Catwalk gait analysis but no improvement in sensorimotor performance (Keuters et al. 2025).

In both ICH studies the behavioral outcomes improved after following interventions targeting meningeal lymphatic functions: cilostazol treatment in the autologous blood injection model significantly improved performance in the cylinder test (Tsai et al. 2022), while rTMS in the collagenase model enhanced neurological and motor functions in the rotarod and the foot fault tests (Y. Liu et al. 2023a, b). In SAH, induced by autologous femoral arterial blood injection, treatments led to improvements in neurological function as measured by the Garcia test (Luo et al. 2024) and MWM (Wang et al. 2022). In GMH, olomoucine treatment exhibited improved short-term neurological performance compared to vehicle-treated controls (Ding et al. 2019). In IVH, photostimulation applied to the skull surface prevented motor impairment by improving NSS, gripping, and forelimb strength, and enhanced memory function (Li et al. 2023).

In a mouse hydrocephalus model, activation of Piezo1, either via transgenic overexpression or treatment with the chemical agonist Yoda1, effectively alleviated the reduced physical activity caused by the disease, as demonstrated by improved performance in open field, rotarod, and mesh hanging tests (Choi et al. 2024).

##### Lymphangiogenic and Morphological Responses of MLVs to Therapeutic Modulation

In MCAO rodent models no significant differences in MLV diameter were observed after CBT (Bai et al. 2022). However, LYVE-1 MLV coverage and diameter increased at 7 days post-stroke following a single i.c.m. delivery of VEGF-C (Boisserand et al. 2024). AAV-VEGF-C significantly increased dural lymphatic vessel area in both dorsal and basal dura mater (Keuters et al. 2025). Cilostazol treatments after ICH resulted in a significant increase in the branching number of MLVs at both left and right transverse sinuses (Tsai et al. 2022). Furthermore, photo-stimulation significantly dilated basal meningeal lymphatic vessels after IVH (Li et al. 2023).

##### Restoring CSF and Meningeal Lymphatic Drainage After Ischemic and Hemorrhagic Brain Injury

Treatments/interventions aiming to enhance MLV drainage were effective across a range of neurological conditions. In ischemic stroke, CBT treatment improved lymphatic inflow and reversed drainage deficits in MCAO rats (Bai et al. 2022). Ischemia itself had a significant effect on clearance of Gd-contrast agent but no striking differences were observed between VEGF-C treated MCAO mice in comparison to control treated ones (Keuters et al. 2025). rTMS enhanced glymphatic clearance and increased tracer transport in both brain and lymphatic tissues after ICH (Y. Liu et al. 2023a, b), while cilostazol promoted lymphangiogenesis and red blood cell (RBC) drainage (Tsai et al. 2022). Dobutamine accelerated CSF and RBC clearance via MLVs in SAH animals, alleviating meningeal congestion (Wang et al. 2022). In SDH models, vitamin D and atorvastatin restored impaired lymphatic drainage by increasing injected dye and RBC transport to the dCLNs (Chen et al. 2024; Yuan et al. 2024). Olomoucine promoted tracer dispersion in the brain parenchyma post-GMH (Ding et al. 2019). Photo-stimulation significantly increased RBC and tracer clearance into dCLNs in both IVH adult mice and neonatal rats (Li et al. 2023). Finally, Piezo1 overexpression in a hydrocephalus model alleviated ventricular enlargement and fluid buildup by restoring brain fluid outflow (Choi et al. 2024).

##### Targeting MLV Function to Suppress Neuroinflammation After Stroke and Hemorrhagic Brain Injury

Emerging evidence suggests that enhancing MLV function may alleviate neuroinflammation across stroke and hemorrhagic brain injury. In a stroke model, VEGF-C pretreatment suppressed microglial activation and pro-inflammatory signaling (including TNF-α/NF-κB and interferon pathways), while promoting non-inflammatory microglial states and neurotrophic signaling, indicating a dual anti-inflammatory and neuroprotective role (Boisserand et al. 2024). Three days after ischemia, VEGF-C-treated mice showed significantly reduced astrocyte activation in the perifocal brain area, while no differences in microglial activation were observed between groups (Keuters et al. 2025). In a rat model of SAH, VEGF-C administration prior to injury reduced levels of cleaved caspase-3 and proinflammatory cytokines IL-1β, IL-6, and TNF-α, suggesting a protective effect through attenuation of apoptosis and inflammation (Luo et al. 2024). In SDH rats, vitamin D treatment significantly decreased TNF-α, IL-6, and IL-8 concentrations in the hematoma and meninges (Chen et al. 2024). At 28 days post-GMH, sustained astrogliosis was evidenced by elevated glial fibrillary acidic protein (GFAP) levels in the brain and increased GFAP-positive cells in the periventricular region, both of which were significantly reduced by olomoucine treatment (Ding et al. 2019).

## Discussion

This systematic review was conducted to gather and critically evaluate preclinical evidence on the therapeutic potential of enhancing meningeal lymphatic drainage across various neurological and neurodegenerative disorders. We aimed to consolidate data from animal studies to assess whether improving brain lymphatic clearance could translate into clinically meaningful interventions.

In this review, we included 30 original preclinical studies using rodent models to explore the therapeutic effects of MLV drainage enhancement on modulating disease pathology, behavior, and neuroinflammation across a spectrum of neurodegenerative and neurological conditions. While studies have previously acknowledged the importance of the glymphatic system and CNS drainage (das Neves et al. 2021; Hablitz and Nedergaard 2021), our analysis highlights the growing recognition of MLVs as critical facilitators of brain waste removal, immune regulation, and recovery from injury.

AD was the most extensively studied condition in this review, accounting for over 40% of all included studies. Our qualitative observations demonstrated that improved lymphatic outflow not only reduces amyloid burden but also modulates neuroinflammation, alleviates other disease-related pathologies and improves behavioral outcomes (Choi et al. 2021; Da Mesquita et al. 2021a, b; J. Li et al. 2024a, b; Lin et al. 2021; Lu et al. 2025; Shan et al. 2024; Wang et al. 2024; Wu et al. 2023; C. Ye et al. 2024a, b; T. Ye et al. 2024a, b). However, not all interventions appeared to be effective. Notably, despite inducing robust dural lymphangiogenesis and improving CSF tracer drainage to dCLNs, VEGF-C failed to reduce hippocampal or cortical Aβ burden, improve behavioral performance, or produce consistent morphological changes in MLVs across three different AD models (J20, APdE9, and 5xFAD, Antila et al. 2024; Da Mesquita et al. 2018). These negative results were attributed to the anatomical mismatch between Aβ deposition sites and the meningeal lymphatic vessels (Antila et al. 2024), and concluded that VEGF-C-mediated lymphatic expansion alone is insufficient in clearing established deep parenchymal Aβ plaques (Da Mesquita et al. 2018).

TBI followed as the second most common focus and interventions to restore MLV function after TBI have yielded encouraging benefits in animal studies. Overall, therapies targeting MLV drainage led to reductions in disease-related pathology such as cerebral edema, improved behavioral outcomes, enhanced CSF clearance, and increased lymphangiogenesis (Dong et al. 2024; Hussain et al. 2023; Liao et al. 2023; Lin et al. 2023; M. Liu et al. 2023a, b).

In the MCAO mouse model of ischemic stroke, impaired meningeal lymphatic development due to a mutation in the kinase domain of VEGFR3 resulted in increased stroke severity measured by larger infarct volumes and greater neurological deficits compared to mice with intact lymphatics (Yanev et al. 2020). In line with these findings, studies included in this systematic review with animal models of ischemic stroke, various types of intracranial hemorrhages and hydrocephalus collectively demonstrated that therapies to enhance the meningeal lymphatic drainage promoted recovery (Bai et al. 2022; Boisserand et al. 2024; Chen et al. 2024; Ding et al. 2019; Li et al. 2023; Luo et al. 2024; Tsai et al. 2022; Wang et al. 2022; Yuan et al. 2024). However, in ischemic stroke, VEGF-C pretreatment did not significantly reduce infarct or edema volumes, and there were no improvements in tracer clearance or alterations microglial activation (Keuters et al. 2025). Moreover, in ICH models, rTMS improved glymphatic clearance and tracer transport but failed to preserve BBB integrity (Y. Liu et al. 2023a, b).

It is well-established that sleep plays a critical role in regulating brain clearance (Hablitz et al. 2020; Lucke-Wold et al. 2015; Rasmussen et al. 2018; Shirolapov et al. 2024). Glymphatic and meningeal lymphatic function are known to be enhanced during sleep, when interstitial space increases and waste removal is more efficient (Yankova et al. 2021). In line with this, one of the studies of this systematic review showed that photobiomodulation facilitated Aβ clearance more effectively during sleep compared to wakefulness (Oxana et al. 2023). This underscores the need to account for the sleep–wake cycle when evaluating therapies targeting meningeal lymphatic clearance, as these physiological states can influence treatment outcomes. In addition, assessing neurological disease-associated sleep disturbances before and after such interventions is essential, given that sleep impairment is a key clinical symptom across all neurological conditions.

Our SYRCLE risk-of-bias assessment revealed several methodological limitations in the reviewed studies. An explanation of whether or how the allocation sequence was generated to produce comparable groups was largely missing. Allocation concealment and random housing were rarely described. Performance and detection bias related to blinding were more frequently addressed. Incomplete outcome data and selective reporting posed concerns in a subset of studies, where discrepancies in group sample sizes across analyses were not explained. Overall, while many studies scored low risk in domains such as baseline characteristics, outcome reporting and other bias (such as in reporting the conflict of interest), inadequate reporting in key areas such as randomization, allocation, and blinding reduces confidence in the robustness of the evidence base.

All in all, the findings suggest that impaired lymphatic drainage is a shared pathological feature across neurological diseases and disorders and therapeutic targeting of this pathway has been shown to reduce pathological protein accumulation, neuroinflammation, and improve functional outcomes. Nonetheless, the limitations such as insufficient reporting should be taken into consideration when interpreting the therapeutic potential of MLV modulation, as they may overstate the beneficial effects or obscure the negative findings.

One of the main limitations of this review is the inability to perform a meta-analysis due to the considerable heterogeneity across studies in terms of animal models, disease types, interventions, outcome measures, and reporting formats. Despite the promising findings, several limitations in the current body of research warrant consideration. First, the heterogeneity in experimental models, intervention timing, and outcome measurements complicates direct comparisons between studies. Most data are limited to young adult male rodents, with scarce exploration of sex, age, or comorbid influences on MLV function.

Currently, there is no animal model that directly demonstrates whether surgical enhancement of lymphatic flow, i.e., deep cervical lymph anastomosis, can influence brain drainage or disease progression. Given the urgent need for clinically relevant, fast-acting interventions, investigating surgical approaches is essential, as they may offer a more immediate and controllable method to restore lymphatic function compared to pharmacological or noninvasive strategies alone. Therefore, future studies should focus on optimizing these clinically feasible strategies and their long-term effects to accelerate translation into human trials, as well as validating these interventions across different diseases, age groups, and sexes to ensure broad and equitable clinical relevance.

## Data Availability

The datasets generated during this systematic review are available from the corresponding author upon request.
